# Dental Education

**Published:** 1899-09

**Authors:** 


### Dental Education.

*Editor Dental Register:*

Permit me to call your attention to, and make a suggestion with reference to dental education. I do not refer to the education of dentists, although this is a prolific source of thought and should not be neglected but constantly pursued till every dentist shall be a thoroughly educated and qualified dental physician and surgeon. But what I wish to refer to is the proper education of the public generally as to the care of the organs of mastication, so as to preserve their health and usefulness through life. I do not propose to present a paper on this subject, but wish to call your attention to its importance, and ask you to give it careful consideration, thorough discussion and take concerted action in the education of the public in general and your patients in particular as to the importance of conserving the health and usefulness of the teeth and mouth from infancy to old age. In order to accomplish this much-to-be-desired result, some means should be adopted to instruct the people and correct their habits. Every child should be placed in the care of an educated, judicious and conscientious dentist as soon as its teeth make their appearance, and that dentist, in connection with the child's parents, should be responsible for the health and usefulness of those teeth during life. It is not enough to require our patients to have their teeth examined occasionally, that any diseased condition may be cured and healed in its incipient stages, but we should instruct them in the fundamental principles of hygiene, that they may supply the organism with the proper elements of nutrition and repair, insuring health at all stages of growth.

We should keep in our own minds and impress upon our patrons the truth that *God can not make good teeth unless we furnish Him the proper material to make them of.* Food containing the necessary elements of nutrition, growth and repair must be furnished to the organism, so that healthy tissues and organs may result and thus fortify every part against disease. This matter of hygiene—the selection of proper food, the proper preparation of that food, the eating properly and living so that it will be thoroughly digested and assimilated—is essential to healthy tissues and sound organs. Although the old adage, "Better late than never," is true in this case, and every one should be instructed to commence now a correct system of hygienic living, yet to secure the best possible results we should teach and never lose sight of the fact that the correct time to commence is, at least, many *months before birth.* Then, as soon as the child is old enough to recognize, he should be introduced to his dentist and learn to look upon him as one of his best friends. The dentist should be familiar with the child, teach him to open his mouth, to allow his gums to be rubbed with the soft ball of the finger, until he learns to enjoy it. So that if disease gets the start of you—as it most likely will—on account of antecedents and habits, your little patient will be proud of himself for submitting to the necessary treatment and operations to restore him to health. Then, by following these principles and practices there would be no tedious and painful operations and your patient would never know the miseries of toothache.

There is another point on which the public should be instructed, viz: that inasmuch as "an ounce of prevention is *worth more* than a pound of cure," therefore preventive instructions and advice should be paid for more liberally than curative treatment and operations. *

